# Supplementary material for: Polyketide synthase gene clusters in the ericoid mycorrhizal fungus Oidiodendron maius: genome-wide prediction, expression profiling, and functional characterization of knockout mutants
Source: Front Plant Sci. 2026 Sep 16;17:1924430. doi: 10.3389/fpls.2026.1924430 (PMC13623903; doi:10.3389/fpls.2026.1924430)
Supplement: Supplementary file 1 [file SupplementaryFile1.zip › Supplementary Materials/Supplementary Folder 1.zip!/Oidma1_AssemblyScaffolds_Repeatmasked/knownclusterblast/cluster49/jgi.p_Oidma1_136692_mibig_hits.html]

| MIBiG Protein | Description | MIBiG Cluster | MiBiG Product | % ID | % Coverage | BLAST Score | E-value |
| --- | --- | --- | --- | --- | --- | --- | --- |
| ALI92654.1 | CitA\_serine\_hydrolase | BGC0001338 | Citrinin\_biosynthetic\_gene\_cluster | 55 | 92.4460431655 | 285 | 8.3e-77 |
| AGN71609.1 | amino\_oxidase/esterase | BGC0000027 | Azaphilone\_biosynthetic\_gene\_cluster | 50 | 99.6402877698 | 269 | 4.7e-72 |
| EHA28230.1 | catalytic\_protein | BGC0001143 | Azanigerone\_biosynthetic\_gene\_cluster | 48 | 93.5251798561 | 240 | 1.8e-63 |
| EAA65600.1 | hypothetical\_protein | BGC0000022 | Asperfuranone\_biosynthetic\_gene\_cluster | 46 | 99.6402877698 | 233 | 3.7e-61 |
| EED18005.1 | conserved\_hypothetical\_protein | BGC0000154 | Stipitatic\_acid\_biosynthetic\_gene\_cluster | 45 | 96.4028776978 | 218 | 7.3e-57 |
| ADB23431.1 | hypothetical\_protein | BGC0000155 | T-toxin\_biosynthetic\_gene\_cluster | 32 | 95.6834532374 | 146 | 6e-35 |
